# HIV-1 pretreatment and acquired antiretroviral drug resistance before tenofovir/ lamivudine /dolutegravir (TLD) roll-out in Mozambique

**DOI:** 10.1186/s12879-024-09579-4

**Published:** 2024-07-29

**Authors:** Nália Ismael, Hernane Gemusse, Isabel Mahumane, Osvaldo Laurindo, Cacildo Magul, Cheryl Baxter, Eduan Wilkinson, L. Marije Hofstra, Nick Wagar, Dulce Bila, Nédio Mabunda, Juliana da Silva, Túlio de Oliveira, Elliot Raizes, Wolfgang Preiser, Pedro Manuel, Artur Ramos, Adolfo Vúbil

**Affiliations:** 1 Instituto Nacional de Saúde, EN1, Bairro da Vila – Parcela N˚3943, Marracuene Sede, Marracuene, Maputo Province, Mozambique; 2 Division of Medical Virology, Faculty of Medicine and Health Sciences, Stellenbosch University, Cape Town, South Africa; 3 Centre for Epidemic Response and Innovation (CERI), School of Data Science and Computational Thinking, Stellenbosch University, Cape Town, South Africa; 4 Division of Global HIV & TB, U.S. Centers for Diseases Control and Prevention, Atlanta, USA; 5 Fundação Ariel Glaser Contra o SIDA Pediátrico, Maputo City, Mozambique; 6 Division of Global HIV & TB, U.S. Centers for Diseases Control and Prevention, Maputo City, Mozambique

**Keywords:** HIV, Drug resistance, Acquired and pre-treatment

## Abstract

**Background:**

The World Health Organization (WHO) recommends that HIV treatment scale-up is accompanied by a robust assessment of drug resistance emergence and transmission. The WHO HIV Drug Resistance (HIVDR) monitoring and surveillance strategy includes HIVDR testing in adults both initiating and receiving antiretroviral therapy (ART). Due to limited information about HIVDR in Mozambique, we conducted two nationally representative surveys of adults initiating and receiving first-line ART regimes to better inform the HIV program.

**Methods:**

We carried out a cross-sectional study between March 2017 and December 2019. Adults (older than 15 years) living with HIV (PLHIV) initiating ART or receiving first-line ART for between 9-15 months at 25 health facilities across all eleven provinces in Mozambique were included. Genotypic HIVDR was assessed on dried blood spots (DBS) when viral loads were  ≥ 1000 copies/ml. Genotypic resistance for non-nucleoside reverse transcriptase inhibitors (NNRTIs), nucleoside reverse transcriptase inhibitors (NRTIs), and protease inhibitors (PIs) was determined using the Stanford HIV database algorithm 9.5 and calibrated population resistance tool 8.1.

**Results:**

Of 828 participants -enrolled, viral load (VL) testing was performed on 408 initiators and 409 ART experienced. Unsuppressed VL was found in 68.1% 419 initiators and 18.8% (77/409) of the ART experienced. Of the 278 initiators and 70 ART experienced who underwent sequencing, 51.7% (144/278) and 75.7% (53/70) were sequenced successfully. Among the new initiators, pretreatment drug resistance (PDR) for NNRTI and PI was found in 16.0% (23/144) and 1.4% (2/144) of the participants, respectively. Acquired drug resistance (ADR) was found in 56.5% (30/53) of the ART-experienced participants of whom 24.5% (13/53) were resistant to both NRTI and NNRTI.

**Conclusion:**

High rates of PDR and ADR for NNRTI and ADR for NRTI were observed in our study. These findings support the replacement of NNRTIs with dolutegravir (DTG) but high levels of NRTI resistance in highly treatment-experienced individuals still require attention when transitioning to new regimens. Moreover, the study underlines the need for routine VL testing and HIVDR surveillance to improve treatment management strategies.

**Supplementary Information:**

The online version contains supplementary material available at 10.1186/s12879-024-09579-4.

## Background

According to the 2023 global report by the Joint United Nations Program on HIV/AIDS (UNAIDS), there has been a substantial decrease in the number of new HIV infections by 57% for southern Africa from 1.2 million in 2015 to 660,000 in 2022 [1]. Widespread access to antiretroviral therapy (ART) has significantly contributed to this success, and by 2022, 29.8 million people living with HIV (PLHIV) were on ART worldwide [2]. Mozambique is located in southern Africa and is highly affected by the HIV epidemic; the estimated treatment coverage in 2022 was 82% in adults with 1.8 out of 2.2 million PLHIV on ART [3]. In 2019–2020, Mozambique transitioned to Dolutegravir (DTG)-based ART regimens, as recommended by the World Health Organization (WHO) [4], and currently 99% of adults are receiving DTG-based regimens [3].

Although the benefits of ART are evident, its rapid scale-up comes with challenges that include retention in care, treatment adherence, and -the emergence of viral strains carrying HIV drug resistance (HIVDR) mutations in treatment-experienced individuals and subsequent transmission of such strains [5, 6]. Acquired (ADR) and transmitted drug resistance (TDR) can compromise viral suppression in patients on ART contributing to ongoing transmission and to morbidity and morbidity. A lack of systematic HIVDR surveillance can lead to the use of suboptimal regimens and put the third 95 target (ensuring 95% of patients on ART have suppressed viral load) at risk with a direct impact on the number of new infections [7].

To detect and monitor the emergence and transmission of HIVDR, the WHO developed surveillance strategies for Low- and Middle-Income Countries (LMICs) [8, 9]. One crucial objective of these strategies is to inform HIV prevention and treatment programs and future therapeutic recommendations. Several countries, particularly LMICs, that conducted HIVDR WHO surveys during 2014-2017 observed pretreatment resistance to nevirapine (NVP) and efavirenz (EFV) above the 10% threshold rate [10, 11]. Following this, in 2018, the WHO recommended the use of dolutegravir (DTG) in first and second-line -ART regimens [12]. DTG is more effective, has a higher genetic barrier, and is more tolerable and easier to take (once daily dose) compared to other ARVs [13]. For Mozambique, a study in Maputo and Tete provinces in 2017 also showed levels of NNRTI-PDR above the 10% threshold, [14] which triggered the country’s decision to transition to tenofovir and lamivudine combined with dolutegravir (TLD) in 2019–2020. Currently, regimens based on DTG are the preferred first-line for both children and adults and the preferred second-line for adults, and currently, 99% of adults are on TLD [3]. Following recommendations by the WHO, we conducted two nationally representative surveys for both Pre-Treatment Drug Resistance (PDR) and Acquired Drug Resistance (ADR).

## Materials and methods

### Study design, population, and enrollment

A cross-sectional survey was conducted in 25 health facilities distributed across all 11 provinces of Mozambique (Supplementary Table 1). The facilities were selected using WHO guidance for sampling ART clinics in countries that combined ADR and PDR surveys [15]. Probability proportional to size (PPS) sampling was used to achieve a nationally representative sample designed to estimate the proportion of adults initiating ART with viral suppression with a precision of +/- 5%. More details about how the desired sample size was computed and how health facilities were selected can be found in Supplementary Information S1. The survey enrolled both ART initiators and ART-experienced adults until a sample size of 16 participants per health facility was achieved for each group, with a total target of 400 participants per group. Patients were considered ART initiators if either ART-naïve or pre-exposed to first-line ART and re-initiating first-line ART after at least 3 months of treatment interruption consistent with the WHO definition [16]. Patients were considered ART-experienced if receiving treatment for 12 months (*+/-* 3 months) at the time of the survey. Consecutive eligible participants were enrolled between March 2017 and December 2019 until the predetermined sample sizes for both groups had been reached for each health facility.

Demographic and clinical information for each patient was collected using a standardized data collection form. Venous blood samples (5 ml) were collected in ethylene-diamine-tetra-acetic acid (EDTA) tubes and dried blood spot samples (DBS) were prepared following WHO guidance [17]. From each participant, five DBS spots, each containing 75 µl of blood, were prepared. All samples were shipped to the *Instituto Nacional de Saúde* located in Marracuene, Maputo Province for further laboratory testing.

### Ethical considerations

The study protocol was approved by the National Bioethics Committee in Mozambique (reference number 123/CNBS/20) and by the Health Research Ethics Committee of Stellenbosch University (reference number S19/10/198). Written informed consent and demographic information were obtained from all participants before blood collection. For participants with difficulty in signing, a fingerprint on the informed consent form was obtained. Individuals aged 15–17 years were also included since they also received the same ART regimens as adults. As these participants are legally considered minors, their caregivers’ (parents or guardians) permission to participate in the study was obtained along with their assent.

### HIV-1 molecular diagnosis and viral load testing

Viral load (VL) testing was performed using one DBS with the COBAS® AmpliPrep/COBAS® TaqMan® HIV-1 Test, v2.0 (Roche Molecular Diagnostics, Branchburg, NJ) kit according to the manufacturer’s instructions. HIV-1 infection was assessed with the COBAS® AmpliPrep/COBAS® TaqMan® HIV-1 Qualitative Test v2.0 [18, 19] kit for all ART initiators with an undetectable VL [18, 19].

### RNA extraction, PCR, and genotyping

HIVDR resistance testing was performed on all samples that had a VL result ≥ 1000 copies/mL according to the WHO/HIV ResNet Laboratory Operational Framework [20] and using a previously published protocol for HIV sequencing using DBS [21]. Total nucleic acid (TNA) was extracted from DBS using the Nuclisens EasyMag platform (BioMérieux, Portugal) according to the manufacturer’s instructions [22]. RNA was first reverse transcribed to cDNA and then amplified by a nested PCR using the HIV-1 Genotyping Kit: Amplification Module version 1.0 (Applied Biosystems, Thermo Fisher Scientific, Foster City, CA, USA). The resulting gene product consisted of the protease (codons 6–99) and reverse transcriptase (codons 1–251) regions of the HIV-1 *polymerase* gene. The amplified product was visualized in 1.0% agarose gel. The sequencing reaction was performed using six primers in the Cycle Sequencing Module of the HIV-1 Genotyping Kit and sequences were generated from 3500XL Genetic Analyzer (Applied Biosystems). Sequences were considered good quality if 90% of the reverse transcriptase and protease regions were successfully sequenced.

### Sequence analysis

Sequences were assembled and edited using the sequence analysis tool ReCall (British Columbia Centre for Excellence in HIV/AIDS, Vancouver, Canada) [23]. Drug resistance mutations (DRMs) and drug susceptibility were interpreted using Stanford HIV Drug Resistance Database Version 8 (Stanford University, California, U.S.A.). To estimate the proportion of sequences with any surveillance drug resistance mutation (SDRM) according to the WHO [24] at a population level, the Calibrated Population Resistance (CPR) tool version 8.1 was used [25]. Analysis was according to the 2009 WHO list of mutations The overall population prevalence HIVDR rate was calculated for each class of ARVs in both groups and only mutations that confer high-level resistance as per the 2009 WHO list were used for analysis. HIV-1 subtype was assigned using BioAfrica REGA HIV-1 automated subtyping tool v3.0 [26] and Jumping Profile Hidden Markov Models [27].

### Statistical analysis

Descriptive analysis included estimation of medians and interquartile ranges (IQR), and proportions with 95% confidence intervals (CI). Prevalence for DRMs and ARVs susceptibility predictions was calculated by dividing the number of patients with the resistance mutations by the total number of participants sequenced for each group.

## Results

### Study population characteristics

**Fig. 1:**
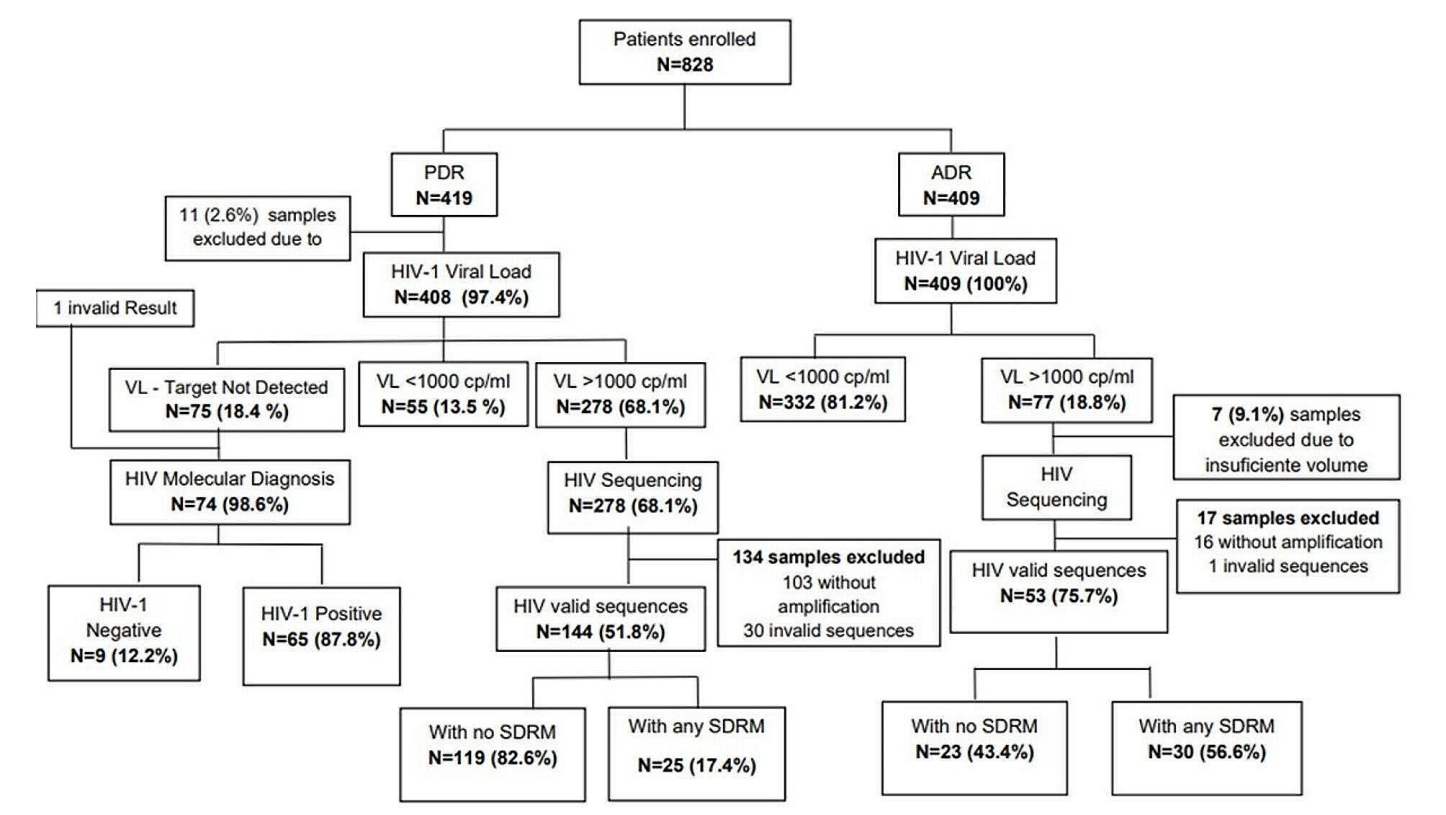
Study flow chart for both ART initiators and ART-experienced participants from 25 health facility in Mozambique 2017–2019, from enrollment to viral load testing, molecular diagnosis, and HIVDR testing. *Abbreviations* PDR, pre-treatment drug resistance, ADR, acquired drug resistance; VL, viral load; SDRM, surveillance drug resistance mutation

A total of 828 participants were enrolled between March 2017 and December 2019, of whom 419 were ART initiators or re-initiators and 409 were ART-experienced. The majority were females, 58.5% (245/419) among the ART initiators and 62.6% (256/409) among the ART-experienced. The median ages of participants were 36 and 32 years for ART initiators and ART-experienced, respectively. Detailed characteristics of the study population are presented in Table 1. Of the 419 ART initiators enrolled, 11 (2.6%) samples were excluded due to insufficient blood and the remaining 408 underwent VL testing (Figs. 1), Of these, 18.4% (75/408) had an undetectable VL (Target Not Detected, TND), 13.5% (55/408) had VL *≤* 1000 cp/ml, and the remaining 68.1% (278/408) had ≥ 1000 copies/ml. For the participants initiating or re-initiating treatment with undetectable VL (*n* = 75), HIV molecular diagnosis was performed in 74 samples of whom one had an invalid VL result and 12.2% (*n* = 9) were HIV-target not detected. Of the ART initiators only 0.7% (3/419) reported previous exposure to ART, with one being exposed to Nevirapine (NVP) for prevention of mother-to-child transmission (PMCT) and the remaining two had been pre-exposed to the first-line ART regimen, TDF + 3TC + EFV (TLE). From the 409 ART-experienced participants that underwent VL testing, 81.2% (332/409) had suppressed viral load according to the 2016 WHO Consolidated guidelines [28] and the remaining 18.8% (77/409) had unsuppressed VL. Of these, seven samples were excluded from sequencing due to insufficient remaining dried blood spots to perform sequencing and the remaining 70 underwent sequencing (17.1%). Samples with successful sequencing rates were found in 51.8% (144/408) among the ART initiators and in 75.7% (53/70) for the ART-experienced.

**Table 1:** Demographic and health facility characteristics of ART-initiators (PDR) and ART-experienced study participants, 2017–2019, Mozambique

Characteristics	ART Initiators (*n* = 419)			ART Experienced (*n* = 409)		
	*n*	Median or %	IQR or 95% CI	*n*	Median or %	IQR or 95% CI
Gender						
Female	245	58.5	(53–63)	256	62.6	(58–67)
Male	173	41.2	(36–46)	150	36.7	(38–41)
No Information	1	0.2	(0–1)	3	0.7	(0–1)
Age in Years						
	419	36	(25–39)	409	32	(26–40)
15–24	102	22	(19–23)	86	21.0	(19–23)
25–44	251	33	(29–37)	267	33	(29–38)
> 45	65	50	(45–52)	56	50	(48–54)
No Information	1					
Year of HIV Diagnosis						
2019	28	6.7	(4–9)	18	4.4	(2–6)
2018	69	16.4	(13–20)	24	5.9	(2–6)
2017	316	75.2	(71–79)	141	34.5	(30–39)
2016	3	0.7	(0–1)	214	52.3	(47–57)
2015				5	1.2	(0–2)
≤ 2014	1	0.2	(0–1)	4	1.0	(0–1)
No Information	2	0.5	(0–1)	3	0.7	(0–1)
Previous Exposure to ARVs						
No	394	94	(91–96)	0	0.0	
Yes	3	0.7	(0–1)	409	100.0	(96–99)
No Information	22	5.2	(3–7)	0		
Current ART Regimen						
TDF + 3TC + EFV				408	99.8	(97–99)
AZT + 3TC + EFV				1	0.2	(0–1)
No Information				0	0.0	
CD4 cells/mm^3^ count median						
< 200	22	130	36–168	14	114	87–121
200–499	35	348	258–413	88	352	294–405
500+	23	741	555–847	82	656	557–851
No Information	339	81.1		225	55	
Viral Load copies/ml median						
≥ 1000	278	12,194	4724–43693	77	400	2665–18510
< 1000	55	493	400–766	47	7654	400–500
TND	75	18		285	70	17
No Information	11	3				

*Abbreviations* IQR, interquartile; CI, confidence interval; ARV, antiretroviral; ART, antiretroviral therapy; VL, viral load; 3TC, lamivudine; ABC, abacavir; AZT, zidovudine; EFV, efavirenz; LPV/r, lopinavir/ritonavir; NVP, nevirapine; TND, target not detected

### HIV subtype analysis

HIV subtype determination was based on the *pol* region sequences (Supplementary Figure S1), with 144 and 53 sequences were generated from ART initiators and ART-experienced patients, respectively. Subtype C was the most frequent subtype with 93.0%(134/144) and 92.4%(49/53). for ART initiators and experienced, respectively Subtype A1 was identified in 6% (9/144) among ART initiators and 2% (1/53) in the ART-experienced group. Mosaic mixture of A1 and D was observed in one participant initiating ART and a mosaic of A1 and C in one ART-experienced participant. Subtypes G and D were only found in ART-experienced participants in 1.9% (1/53) each.

### Drug resistance mutations and susceptibility in newly initiating patients

**Fig. 2:**
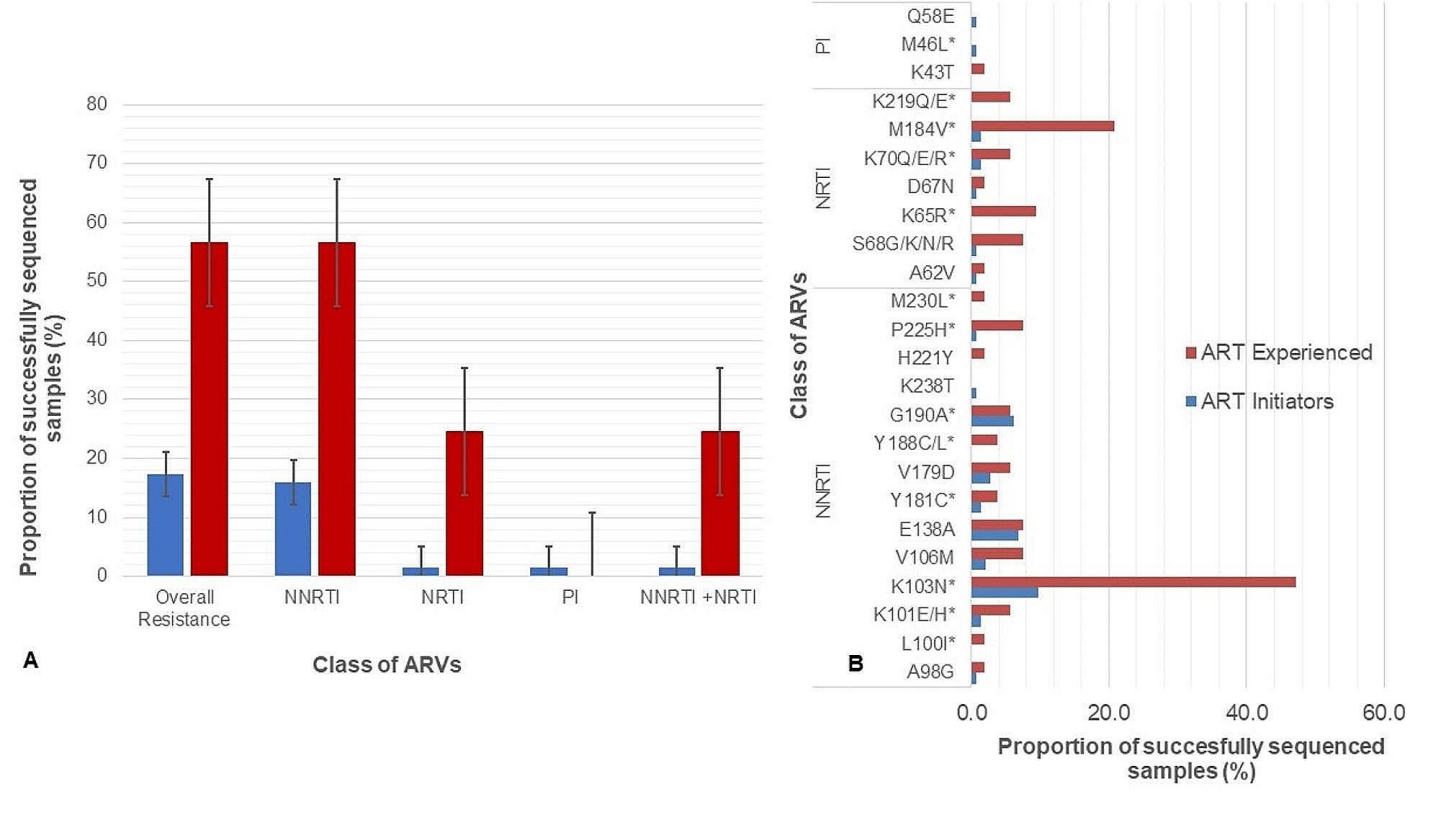
Pre-treatment (PDR) and acquired drug resistance (ADR) pproportion of successfully sequenced samples and mutation pattern profiles, 2017–2019, Mozambique. (**A**) Proportion of sequences among the ART initiators and ART-experienced with any surveillance drugs resistance mutation (SDRMs) to the different classes of ARVs. Prevalence was determined using the calibrated population resistance (CPR) version 8.1,. (**B**) PDR mutation profile prevalence rate for the different class of ARVs, *major drug resistance mutations, ^TAM, thymidine analogue mutations

**Fig. 3:**
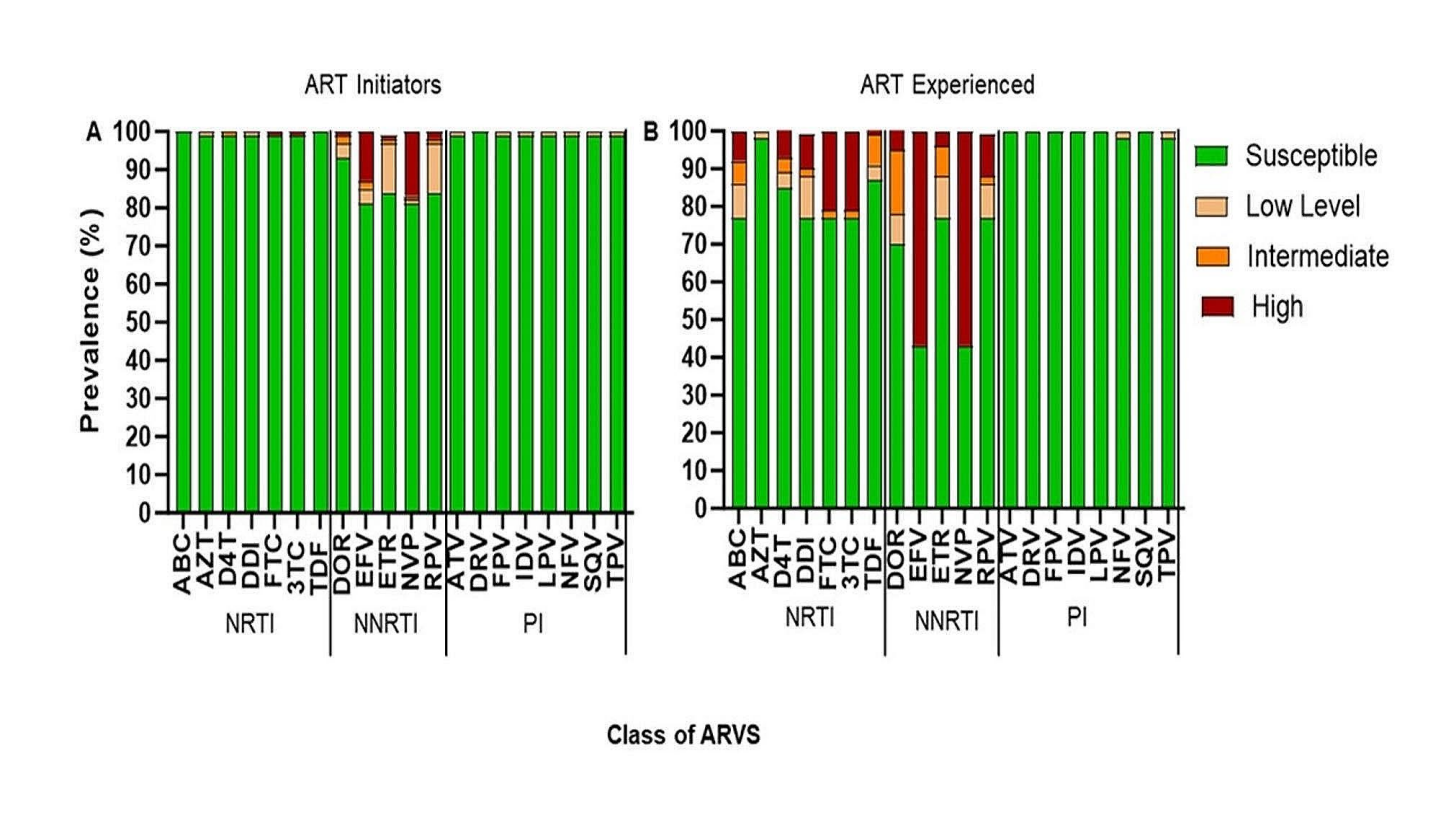
Predicted resistance to individual antiretroviral drug among viremic ART initiators and ART-experienced participants, 2017–2019, Mozambique. Resistance is classified as per NNRTI, NRTI, PI scoring algorithms from the Stanford HIV Drug Resistance Database Version 8.8.0. *Abbreviations* ARV, antiretroviral therapy; NRTI, nucleoside reverse transcriptase inhibitors; NNRTI, non-nucleoside reverse transcriptase; INSTI, integrase strand transfer inhibitor; ABC, abacavir; AZT, azidothymidine (zidovudine); D4T, stavudine; FTC, emtricitabine; 3TC, lamivudine; TDF, tenofovir; DOR, doravirine; EFV, efavirenz; ETR, etravirine; NVP, nevirapine; RPV, rilpivirine; ATV, atazanavir; DRV, Darunavir; LPV, lopinavir; TPV, tipranavir

Twenty-five initiators had at least one Surveillance Drug Resistance Mutation (SDRM) representing an overall PDR of 17.4% (95% CI 11–24). NNRTI and PI resistance mutations were observed in 16.0% (23/144) (95% CI 10–22) and 1.4% (2/144) (2, 95% CI 0–3), respectively (Fig. 2a). The most common NNRTI resistance mutations were K1013N (14/144, 9.7%), followed by polymorphic mutation E138A with 6.9% (10/144) and major mutation G190A with 6.2% (9/144) (Fig. 2b). For NRTI resistance mutations, non-thymidine analog mutations (non-TAMs) that prevent NRTI incorporation were detected in our study. M184V which confers high-level resistance to lamivudine (3TC) was the most common non-TAM with 1.3% (2/144). Moreover, thymidine analog mutation (TAMs) D67N with 0.7% (*n* = 1) and K70Q/E/R with 1.3% (2/144) were also observed. The PI resistance mutations M46L and Q58E, which are known to be associated with reduced susceptibility to atazanavir (ATV) and lopinavir (LPV), were observed in two participants with 0.7% (1/144) . When considering individual drug susceptibility prediction, high-level resistance for Efavirenz (EFV) and NVP was detected in 13.1% (19/144) and 16.6% (24/144) respectively (Fig. 3). There was no intermediate to high-level resistance for tenofovir disoproxil fumarate (TDF) among the new initiators, but 1.3% (2/144) of the participants showed high-level PDR to 3TC. Low proportions below 5% among the initiators showed intermediate to high-level resistance to etravirine (ETR) a second-generation NNRTI and Rilpivirine (RPV) that is used in combination with cabotegravir (CAB) as a long-acting INSTI and NNRTI combination ARV.

### Drug resistance mutation and susceptibility for the -ART-experienced

Among the ART-experienced participants with viral load ≥ 1000 copies/ml and good quality sequences (*n* = 53), 30 had at least one SDRM representing an overall prevalence of resistance of 56.6% (30/53) (95% CI 42–70) (Fig. 2a). Mutations that confer resistance for both NRTI and NNRTI were observed in 24.5% (13/53). The most common NNRTI resistance mutation was K103N with 47.2% (*n* = 25/53), followed by P225H with 7.5% (4/53) and G190A with 5.6% (3/53) (Fig. 2b). All mutations mentioned above (K103N, G190A, P225H) are associated with high-level resistance to EFV and NVP. Polymorphic mutation E138A was also prevalent, with 7.5% (4/53). NRTI resistance mutation M184V which confers high-level resistance to lamivudine (3TC) was the most common in this group with 20.7% (11/53). K65R resistance mutation associated with high-level resistance to TDF and abacavir (ABC) was also found in 9.4% (5/53). Furthermore, low levels of TAMs (D67N and K70Q/E/R) and K219Q/E with 5.6% (3/53) were detected. High-level resistance to EFV and NVP was observed in 56.6% (30/53) (Fig. 3). Intermediate to high-level resistance for 3TC and tenofovir (TDF) was observed in 22.6% (12/53) and 9.4% (5/53) participants, respectively. For the second-generation NNRTIs, intermediate to high-level resistance was observed in 7.7% (6/53) for ETR and in 12.3% (7/53) for RPV.

## Discussion

Our study confirms findings from other PDR and ADR surveys conducted in Maputo and Tete [14] provinces of Mozambique and several other studies in Southern and Eastern Africa reporting PDR and ADR to NNRTI that exceeds 10% [29–31]. In our study PDR and ADR were primarily driven by the K103N mutation which causes over 20-fold increased resistance to EFV [32] and is slower to revert than other mutations with a viral fitness similar to wild type [33]. Most of the ART initiators in our study were susceptible to TDF and 3TC, and only one participant presented high-level resistance to 3TC. Altogether, these findings support the transition from NNRTI-first line-based regimens to TLD for people initiating treatment. Besides this, DTG is also known to be a very potent ARV, with fewer side effects, more tolerable, and with a higher genetic barrier. In addition, DTG can be used in combination with other nucleoside ARVs as a first-line treatment regimen in a single tablet form used once daily [34, 35].

Thus, among the viremic ART-experienced participants, a relatively high proportion of NRTI resistance mutation M184V/1 above the 10% threshold and K65R close to 10% associated with increased resistance to 3TC and TDF were detected. Both ARVs compromise the recommended TLD regimen backbone. Although mutation M184V/I provides an advantage to the virus in the presence of 3TC, it comes at a fitness cost, which means that the replication capacity of the virus is reduced compared to the wild type [35]. While recent findings show successful treatment outcomes for patients harboring NRTI mutations after transitioning to DTG-based regimens, there are still conflicting data about the real long-term impact of pre-existing NRTI resistance mutations on patients who transitioned to TLD from previous first-line treatment with TLE after the rapid roll-out in LMICS [36–38]. As reported before [37], resistance to 3TC and TDF can result in DTG functional monotherapy and subsequent emergence of DTG resistance. Our results highlight the need to closely monitor patients on TLD who have experienced virological failure with previous first-line regimens, such as TLE. Therefore, more research addressing this in LMICs is needed as resistance to NRTI in highly treated patients experiencing virological failure is common in these settings. These results underline the need to better monitor patients with a history of virological failure from previous treatment regimens that have transitioned to TLD.

High PDR levels observed in our study among those initiating treatment suggest that transmission of HIV strains resistant to EFV and NVP has been happening in Mozambique which might have jeopardized previous first-line ART regimen outcomes [39]. The presence of some mutations such as Y181C and E138A among the initiators and ART experienced may in future have an impact on second-generation NNRTIs, such as ETR and long-acting CAB/RPV [40] Such findings emphasize the need for continued surveillance to better inform programs on new treatment strategies such as second-generation NNRTIs, particularly in LMICs.

Even though Mozambique still faces challenges in achieving the UNAIDS 95-95-95 target, the prevalence of viral load suppression (VLS) increased from 81.2% in 2017 from our study and to 88% in 2019 according to the national HIV program of report of Mozambique [3]. Such improvements may be associated with the introduction of TLD in 2019, but HIVDR can still put the third UNAIDS target at risk. Therefore, systematic VL testing, improved ART failure management, and periodic resistance monitoring at a population level are vital in achieving the third target.

Among the new ART initiators, 18.4% had an undetectable VL, of which 12.2% had a subsequent HIV-1 target not detected with a molecular test. The high proportion of undetectable VL tests is surprising given that in the absence of ART, control of viral replication to below 50 copies/ml, known as elite control, is rare albeit possible possibly [41]. Another explanation for this would be undisclosed ART use or misinterpretation of results when two consecutive tests are used [42]. Such false diagnosis can lead people who are not infected being enrolled in ART. This finding underscores the need for ongoing research of such phenomena as well as monitoring of testing procedures and strategies to minimize such errors, particularly in settings with high HIV burden and under-resourced staff where mistakes can easily happen. Moreover, our result also shows the under-reporting of ART use in our study resulting in misclassifying ART-experienced with ART initiators most likely driven by stigma [43], or as a result of integrity issues of DBS samples that might have affected our results.

## Study limitations

Our study has several limitations. Limited resources for patient enrollment and data collection hampered the questionnaire data’s quality and accuracy, and it took longer than expected to achieve the desired sample size. As a result, potentially important factors that include social, demographic, and clinical (e.g. number of sex partners, employment status, history of tuberculosis) information that could contribute to a more comprehensive understanding of clinical and demographic factors associated with PDR and ADR at a population level were overlooked because most of the questions in the questionnaires were not addressed at the sites as initially planned. Further, prior exposure to ART was based on verbal reporting and patient file information, which might have resulted in the inadvertent inclusion of undisclosed ART-experienced patients, as evidenced also by the undetectable VL results among some ART initiators. Fourthly, a sequencing success rate below 80% was observed in both the ART initiators and the ART-experienced participants, which can be partially explained by the sample type used. While well-collected and properly stored DBS samples provide similar yields compared to plasma samples, ideal DBS conditions are rarely met in low-resource settings where they have been confirmed to have a lower sequencing success rate compared to plasma [44]. The long delays until sample pick-up from health facilities may also have influenced sample quality. While our sample was designed to be representative of the patient population in Mozambique, we are presenting unweighted results rather than population estimates. Lastly, the low sample size for the ADR being analyzed might have not truly represented the actual resistance.

## Conclusions

In summary, we found concerning evidence of pre-treatment NVP and EFV resistance, which supports the transition from an NNRTI-based regimen to DTG in Mozambique. However, high-level acquired NRTI resistance suggests a need for urgent attention for patients with previously confirmed virological failure when transitioning to DTG since the risk of failure is higher. To safeguard current treatment options in Mozambique, rigorous VL monitoring and periodic HIVDR surveillance in both ART initiators and ART-experienced patient populations is vital to fill in programmatic gaps and design public health strategies. These gaps can promote the emergence of HIVDR which can hinder the efficacy of future ART regimens and compromise current UNAIDS targets.

### Electronic supplementary material

Below is the link to the electronic supplementary material.

Supplementary Material 1

## Data Availability

All the data generated or analyzed during this study are included in this published article sequences are available in GenBank with accession numbers: PP511921- PP511973, PP512312- PP512455.
