# Supplementary material for: HIV-1 pretreatment and acquired antiretroviral drug resistance before tenofovir/ lamivudine /dolutegravir (TLD) roll-out in Mozambique
Source: BMC Infect Dis. 2024 Jul 29;24:748. doi: 10.1186/s12879-024-09579-4 (PMC11285440; doi:10.1186/s12879-024-09579-4)
Supplement: Supplementary file 1 — Supplementary Material 1 [file 12879_2024_9579_MOESM1_ESM.docx]

**Supplementary Information S1**

**Sample design:**

Health facilities were selected following the World Health Organization ResNet methodology for designing an ADR/PDR survey (26). Specifically, we selected sites using probability proportional to size (PPS) sampling, from a list of all health facilities in the country that provided antiretroviral treatment (ART). The measure of size was based on the number of adults on ART obtained from routinely-reported data. Consecutive eligible patients initiating ART or currently on ART were enrolled until the predetermined sample size for each health facility was achieved for each survey being implemented. Sampling was stratified by geographic region with a proportional distribution of the total number of health facilities.

### Sample size calculations:

For the sample size calculations, included formulas, assumptions, and conditions advised by the WHO in their guidance documents (20). Please refer to these documents for a detailed description of these assumptions.

**The following assumptions were used to calculate the sample size for Adult PDR surveys:**

- Prevalence of HIVDR among all ART initiators: 10%
- Estimated proportion of ART initiators with prior exposure to ARVs: 25%
- Genotyping failure rate: 20%
- Polymerase chain reaction (PCR) amplification rate: 80%
- Intracluster Correlation Coefficient (ICC) for HIVDR outcome: 1%
- Design Effect (DEFF) for PPS sampling: 1.1

**The following assumptions were used to calculate the sample size for the ADR surveys:**

-For adults receiving ART for 12 (+/- 3) months:

- Prevalence of Viral load suppression: 85%
- Polymerase chain reaction (PCR) amplification rate: 80%
- Intracluster Correlation Coefficient (ICC) for HIVDR outcome: 0.004
- DEFF for PPS sampling: 1.1 (note the WHO guidance employs PPS sampling, with DEFF = 1.5)

The sample size was the number of ART initiators sampled per clinic and was calculated as follows:

m_samp_ = m/(R_g_ * I_ARV_)

Where

R_g_ is the genotyping success rate, which is assumed to be 80%

I_ARV_ is the proportion of initiators without prior ARV exposure, assumed to be 75%

m is the unadjusted number of initiators sampled per site and is calculated as:

**Where**

n is the number of sites to be collected.

ICC_DR_ is the intracluster correlation for the drug resistance outcome, assumed to be 0.004 as recommended by WHO guidance

DEFF_info_ is the design effect due to using imperfect data for sampling calculations, assumed to be 1.5

N_eff_ is the number of patients to be sampled and is calculated as:

N_eff_ = (t _df,0.975_)^2^ P_DR_ (1-P_DR_)

L^2^

Where

t =degrees of freedom (# sites -1 )

P_DR_=assumed prevalence of drug resistance among all ART initiators

L=half length of confidence interval

The order of calculations is as follows:

1. N_eff_
2. m
3. m_samp_

Example of a sample size calculations for 20 sites:

1. N_eff_

N_eff_ = (t_19, 0.975_)^2^0.10(1-0.10) / 0.05^2^

N_eff_ = 2.093^2^*0.10*0.90 / 0.05^2^

N_efff_ = 157.7

Neff = 158

1. m

12.4

1. m_samp_

m_samp_ = 12.4/(0.80 * 0.75)

m_samp_ = 20.6

m_samp_ = 21

To achieve adequate statistical power a total of 420 initiators was estimated, which included 20 health facilities with 21 initiators for each.

The same formulas were to calculate sample size for Adult PDR surveys However, assumptions were different.

For Adult ADR at 12 months a prevalence of viral load suppression of 85% was assumed and the proportion of patients with prior ARV exposure was assumed to be 95%. Therefore the sample size was calculated as follows:

1. N_eff_

N_eff_ = (t_19, 0.975_)^2^0.85(1-0.85) / 0.05^2^

N_eff_ = 2.093^2^*0.85*0.15 / 0.05^2^

N_efff_ = 223.4

N_eff_ = 224

1. m

17.9

1. m_samp_

m_samp_ = 12.4/(0.80 * 0.95)

m_samp_ = 23.6

m_samp_ = 24

To achieve adequate statistical power, 480 patients were estimated, including 20 health facilities with 24 patients in each.

**Determination of number of sites:**

The number of sites:

The patient sample size determined above was the *minimum* number of patients to include because the number of patients selected per health facility was the same for each health facility in both surveys (ADR and PDR).

Very small sites were excluded from sampling if they, in total, did not contribute more than 10% of the total population of ART patients. In addition, it was impossible to determine with complete assurance if a health facility was able to support the sample size or otherwise implement the survey. Therefore, to account for the possibility of site dropout, an additional buffer of ~20% of sites was included, with the number collected per site corresponding to the number required for the effective sample size (assuming 20% of the sites are unable to implement the survey).

*Example: For the PDR and ADR, Mozambique calculated that the target patient sample size was 320, collected from 20 sites (16 per site). To include the 20% buffer in site participation, the PPS sampling frame includes 20/0.80 = 25 sites. From each of these 25 sites, it was estimated to enroll 16 patients per site. This ensures that if 20% of the sites are unable to participate, the target sample size of 320 will still be reached. If all sites participate, the sample size will exceed the target, resulting in greater statistical power. The PPS sample for the PDR survey was included below and is an example of the PPS samples that was calculated.*

| Table S1: PPS table with sites randomly selected for inclusion in PDR (n=25), stratified by geographic region. The same sites were also used for the ADR survey | | | | |
| --- | --- | --- | --- | --- |
| **Region** | **Province** | **District** | **Site** | **Number of Initiators** |
| NORTHERN | NAMPULA | ERATI | NAMAPA | 529 |
| NORTHERN | NIASSA | MECANHELAS | MECANHELAS | 335 |
| NORTHERN | NAMPULA | MOGOVOLAS | NANHUPO RIO | 120 |
| NORTHERN | CABO DELGADO | ANCUABE | ANCUABE CS | 277 |
| NORTHERN | NAMPULA | ERATI | SAMORA MACHEL | 95 |
| NORTHERN | NAMPULA | MURRUPULA | TIPONHA | 144 |
|  |  |  |  |  |
| CENTRAL | MANICA | GONDOLA | AMATONGA | 242 |
| CENTRAL | SOFALA | CIDADE DA BEIRA | SAO LUCAS | 339 |
| CENTRAL | ZAMBEZIA | MILANGE | DULANHA | 191 |
| CENTRAL | TETE | MACANGA | MACANGA | 103 |
| CENTRAL | TETE | ANGÓNIA | DÓMUE | 96 |
| CENTRAL | ZAMBEZIA | MILANGE | DACHUDUA | 124 |
| CENTRAL | MANICA | MANICA | MESSICA | 369 |
| CENTRAL | TETE | CHANGARA | MSAUA | 182 |
| CENTRAL | ZAMBEZIA | MORRUMBALA | DERRE | 113 |
| CENTRAL | MANICA | TAMBARA | NHACOLO | 149 |
| CENTRAL | TETE | CAHORA BASSA | CHIRODZI-PONTE | 80 |
|  |  |  |  |  |
| SOUTHERN | GAZA | DISTRITO DE XAI-XAI | SIAIA | 126 |
| SOUTHERN | GAZA | CHIBUTO | MUXAXANE | 106 |
| SOUTHERN | MAPUTO PROVINCIA | MARRACUENE | RICATLA | 158 |
| SOUTHERN | GAZA | CHIBUTO | ALTO CHANGANE | 87 |
| SOUTHERN | GAZA | DISTRITO DE XAI-XAI | CHICUMBANE | 886 |
| SOUTHERN | MAPUTO CIDADE | KAMAXAKENE | CS 1º DE MAIO | 1308 |
| SOUTHERN | INHAMBANE | CIDADE DE INHAMBANE | BALANE (URBANO) | 609 |
| SOUTHERN | GAZA | CHIBUTO | CELULA MISSAVENE | 98 |

**Supplementary Figure S1**

**
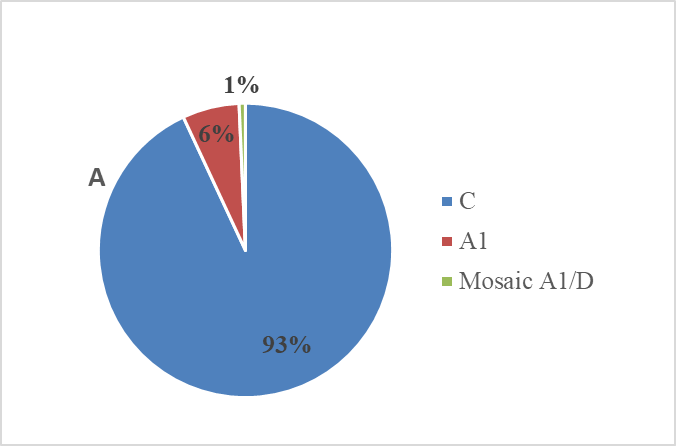

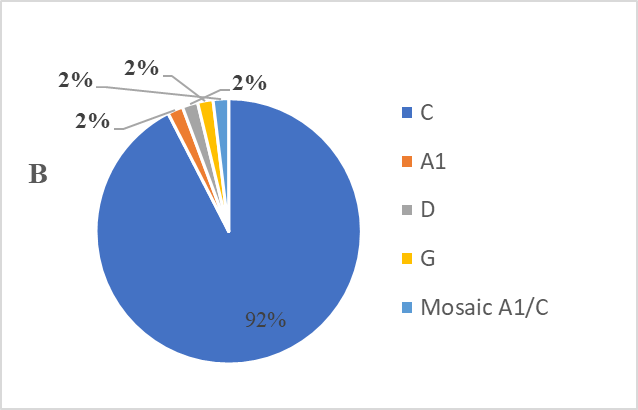
**

**Figure S1:** Distribution of subtypes among the A) ART initiators and B) ART experienced
